# Increased arterial stiffness parameters in panic disorder patients in long term treatment period

**DOI:** 10.1186/s12991-016-0102-6

**Published:** 2016-06-08

**Authors:** Omer Yanartas, Murat Sunbul, Zeynep Senkal, Erdal Durmus, Tarik Kivrak, Nilufer Subasi, Gulhan Karaer, Serhat Ergun, Ibrahim Sari, Kemal Sayar

**Affiliations:** Department of Psychiatry, Marmara University School of Medicine, Istanbul, Turkey; Department of Cardiology, Marmara University School of Medicine, Istanbul, Turkey

**Keywords:** Anxiety, Depression, Arterial stiffness, Pulse wave velocity, Symptom severity

## Abstract

**Background:**

The relationship between mental stress and cardiovascular disease has been shown in several studies. Panic disorder (PD) is also associated with cardiovascular disease due to increased risk of myocardial infarction. The aim of this study is to evaluate the association between arterial stiffness parameters and depression/anxiety scores in patients with PD.

**Methods:**

The study population consisted of 25 patients with PD and 25 age–sex-matched healthy controls. Depression and anxiety levels were evaluated by Beck Depression Inventory (BDI) and Beck Anxiety Inventory (BAI), respectively. Determination of arterial stiffness parameters was conducted using a Mobil-O-Graph arteriograph system that detected signals from the brachial artery.

**Results:**

While baseline characteristics were similar between two groups, BDI and BAI scores were significantly higher in patients with PD (*p* < 0.005). The pulse wave velocity (PWV) and Augmentation Index (AIx) were also significantly higher in patients with PD (*p* = 0.001, *p* = 0.006). There was a moderate correlation between PWV and AIx with BAI scores (*r* = 0.442, *p* = 0.001, *r* = 0.441, *p* = 0.001). AIx was also positively correlated with BDI scores (*r* = 0.415, *p* = 0.03).

**Conclusion:**

We demonstrated a significant relationship between arterial stiffness parameters and anxiety/depression scores in patients with PD who receive antidepressant treatment.

## Background

Cardiovascular disease, anxiety and depression are among the most prevalent health problems [1]. Previous studies suggest that; although vascular health is mainly determined by age, acute and chronic mental stress may also cause cardiovascular disease [2, 3]. Epidemiologic studies have shown that patients with panic disorder, a subtype of anxiety disorder, have higher risk of fatal myocardial infarction and sudden cardiac death compared to normal population and also the risk of a fatal event increases in higher levels of anxiety [4, 5]. Arterial stiffness is one of the major signs of vascular aging, which has been documented as a prominent independent prognostic factor of cardiovascular mortality [6, 7]. Progressive loss of arterial elasticity may cause important complications such as stroke, left ventricular hypertrophy, and hypertension [7]. Therefore, arterial stiffness has increasingly drawn attention for its role in the cardiovascular disease (CVD) [8]. Increased arterial stiffness was linked with systemic inflammation in patients with psoriasis [9]. In a study anxiety was associated with cardiovascular diseases and it was hypothesized that inflammation has a role in that association [10]. Thus, inflammation may have critical role between arterial stiffness and anxiety.

Previous studies have demonstrated that depression and anxiety may play an important role in vascular aging [5, 11, 12]. Cicek et al. [11] have found that increased pulse wave velocity is related to panic disorder. In addition to the presence of an anxiety disorder, the positive correlation between the severity of anxiety symptoms and vascular stiffness parameters has also been demonstrated in the study, whereas the frequency of panic attacks and its relation to arterial stiffness have not been investigated [11]. The comorbidity of anxiety and depressive disorders has been shown to be a distinct risk factor for increased arterial stiffness parameters, respectively [12].

The usage of antidepressant medication may have effects on cardiovascular physiology but its relation to arterial stiffness is unclear. In three studies both decreasing symptom severity and using of antidepressant medication have been found to be related to arterial stiffness [8, 13, 14].

The aim of this study is to evaluate the association between arterial stiffness parameters and depression/anxiety symptom severity in patients with panic disorder.

## Methods

### Subjects

The study was carried out between March and December 2013 in psychiatry outpatient clinics at Marmara University Hospital. Twenty-five patients with panic disorder according to the criteria of Diagnostic and Statistical Manual of Mental Disorders, Revised Text (DSM IV, TR; APA 2000) and 25 age–gender matched healthy controls, not having any treatment, were enrolled in the study, respectively. Patients were excluded from the study if they met one of the following criteria: (1) age <18 and >65 years, (2) illiteracy, (3) having extra-cardiac risk factors such as: past or current hypertension (systolic and/or diastolic blood pressure ≥140/90 mmHg), diabetes mellitus (fasting plasma glucose levels more than 126 mg/dL in ≥3 measurements), hyperlipidemia (serum total cholesterol ≥200 mg/dl, serum triglyceride ≥150 mg/dl, low-density lipoprotein cholesterol ≥130 mg/dl) or use of antihypertensive, antidiabetic or lipid-lowering medication, (4) history of nicotine, alcohol or substance abuse/dependence, (5) having any psychiatric comorbidity related to more chronic vulnerability to stress and anxiety such as; generalized anxiety disorder, major depressive disorder were excluded from the study. Eight patients who had comorbid depression and five patients who had comorbid anxiety disorder have been excluded from the study. Since antidepressant treatment varieties and antidepressant treatment period may effect the stiffness parameters we included patients to our study who takes only SSRI (selective serotonin reuptake inhibitor) medication. In panic disorder treatment at least 12–14 month is recommended as maintenance treatment [15]. Thus, in this study we included the patients having antidepressant treatment at least 12 months. Frequency of panic attacks was assessed by asking patients to estimate the number of panic attacks they experienced in the last month.

All patients signed a written informed consent prior to the study. The study was conducted in accordance with the principles stated in the Declaration Criteria of Helsinki and approved by the Local Ethics Committee of Marmara University.

### Procedure

#### Assessment of arterial stiffness parameters

All participants were refrained from eating and drinking alcohol, coffee, or tea for at least 12 h prior to the study. The test of arterial stiffness was performed in the supine position in a quiet, temperature-controlled room (22–24 °C) in the early morning hours (between 08.00 and 10.00 a.m.). Measurements were carried out using a Mobil-O-Graph arteriograph system (Mobil-O-Graph NG, Stolberg, Germany) [16]. This system detects signals from the brachial artery even though cuff pressure is 35 mmHg higher than systolic pressure in the brachial artery. The technique is based on the fact that the contraction of the myocardium initiates a pulse wave (early systolic peak) running down in the aorta [16]. This first wave is reflected from the aortic wall at the distal branching point and causes a reflected second wave (late systolic peak). The morphology of this second reflected wave depends on the stiffness of the large artery. Using amplitude and time difference of the first and the second wave, Augmented Index (AIx adjusted for heart rate 75 bpm) and pulse wave velocity (PWV) are calculated according to current guidelines [16]. The doctor measuring PWV was unaware of the psychometric results of the study population.

#### Psychometric assessment

Before all participants were asked to fulfill Beck Depression and Beck Anxiety Inventory, brief-sociodemographic questionnaire was filled by the clinician. The Beck Depression Inventory (BDI) measures factors linked with the affective (e.g., hopelessness, irritability, cognitive problems, feelings of guilt or being punished) and somatic (e.g., fatigue, weight loss, and lack of sexual desire) constituents of depression. The BDI has 21-item and cut-off point for that scale is 21 [17, 18].

The Beck Anxiety Inventory (BAI), which is a self report questionnaire, measures anxiety scores and cut-off scores are <7 minimal anxiety, 8–15 mild anxiety, 16–25 moderate anxiety, and 26–63 severe anxiety [18]. The validity and reliability studies of both BDI and BAI for adaptation to the Turkish language have been performed [19, 20].

### Statistical analysis

Statistical analyses were performed using SPSS 20.0 statistical package for Windows. Continuous data were expressed as mean ± standard deviation while categorical data were presented as percentage. Chi-square test was used for comparison of categorical variables while student *T* test or Mann–Whitney *U* test were used to compare parametric and nonparametric continuous variables, respectively. Normality of distribution was assessed by Shapiro–Wilk test. Correlation analysis was performed using Spearman’s correlation test. Linear regression analyses were performed to determine the predictors of BAI and BDI scores. A value of *p* < 0.05 was considered to be statistically significant.

Effect size was defined according to effect size guide for clinicians and researchers [21]. We calculated effect sizes of BDI, BAI, PWV, and Aix. Effect sizes of BDI, BAI, PWV, and AIx were 1.6, 2.9, 1.1, and 0.8, respectively. Our results demonstrated that those parameters had large or very large effect size.

## Results

Twenty-five consecutive patients with panic disorder (19 female and 6 male, mean age: 34.8 ± 7.9 years) and 25 healthy volunteers (16 female and 9 male mean age: 35.5 ± 7.5 years) were included in the study. Baseline characteristics and clinical data of study population are shown in Table 1. While demographic data of study population was similar between two groups, BDI and BAI scores were significantly higher in patients with panic disorder compared to controls (*p* < 0.001).

**Table 1:** Comparison of baseline characteristics and clinical data of the study population

	Patient with panic disorder (n = 25)	Control group (n = 25)	*p*
Age^a^ (years)	34.8 ± 7.9	35.5 ± 7.5	0.756
Female gender^b^, *n* (%)	19 (76)	16 (64)	0.355
Body surface^d ^area^a^ (m^2^)	1.86 ± 0.26	1.80 ± 0.23	0.392
BDI scores^c^	17.8 ± 9.6	5.9 ± 5.2	*<0.001*
BAI scores^c^	31.5 ± 13.8	6.3 ± 3.6	*<0.001*
Antidepressant treatment	Paroxetine (n = 9)		
	Escitalopram (n = 7)		
	Sertraline (n = 5)	–	
	Fluoxetine (n = 3)		
	Fluvoxamine (n = 1)		

Italic values indicate statistically significant (*p* < 0.05)

^a^Student *T* test was used

^b^Chi square test was used

^c^Mann–Whitney *U* test was used

^d^Data are presented as mean ± standard deviation or number of patient

**Fig. 1:**
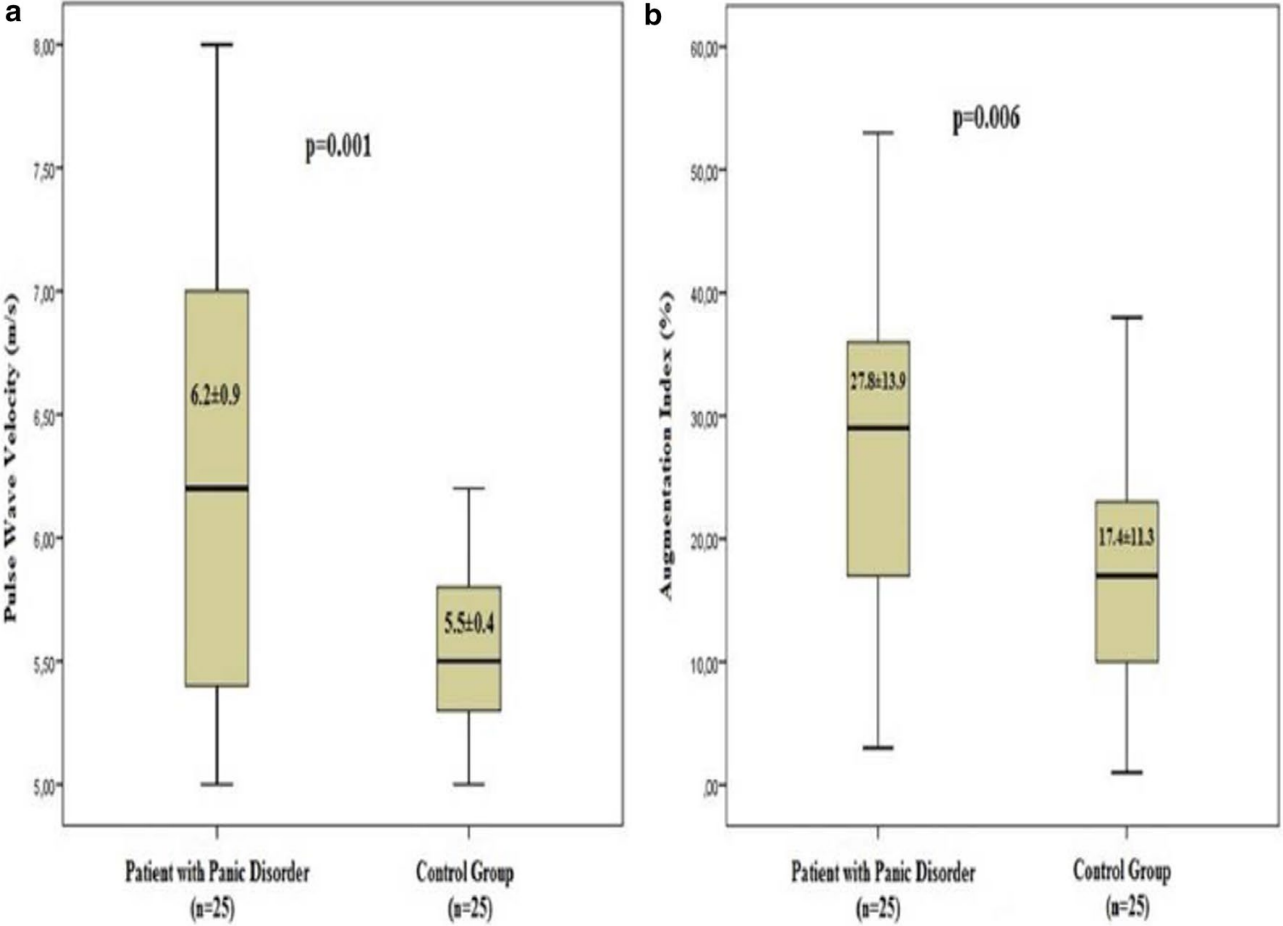
Comparison of pulse wave velocity and Augmentation Index values of the study population

**Fig. 2:**
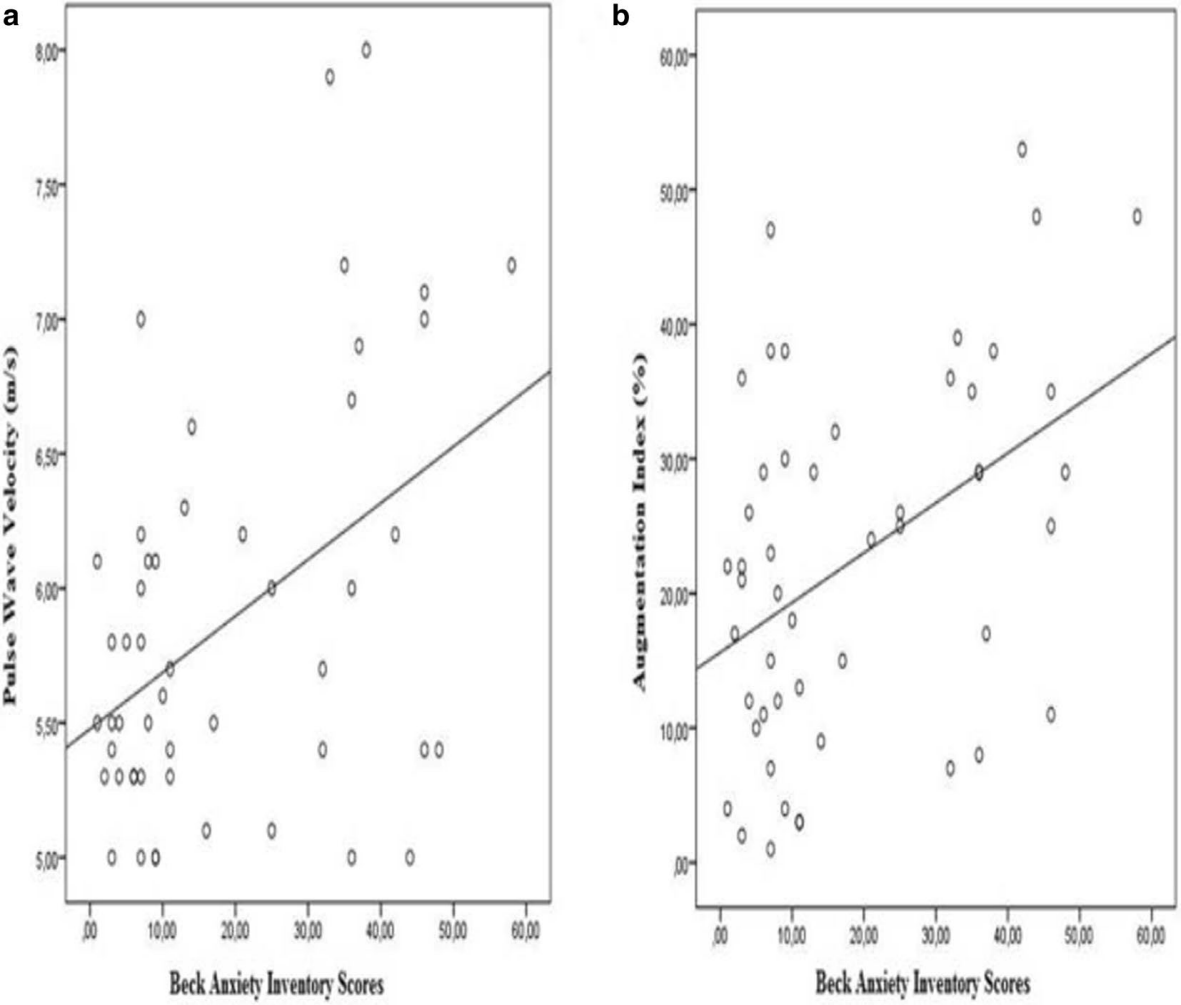
Correlation analysis of Beck Anxiety Inventory scores with pulse wave velocity (**a**) and Augmentation Index (**b**)

**Fig. 3:**
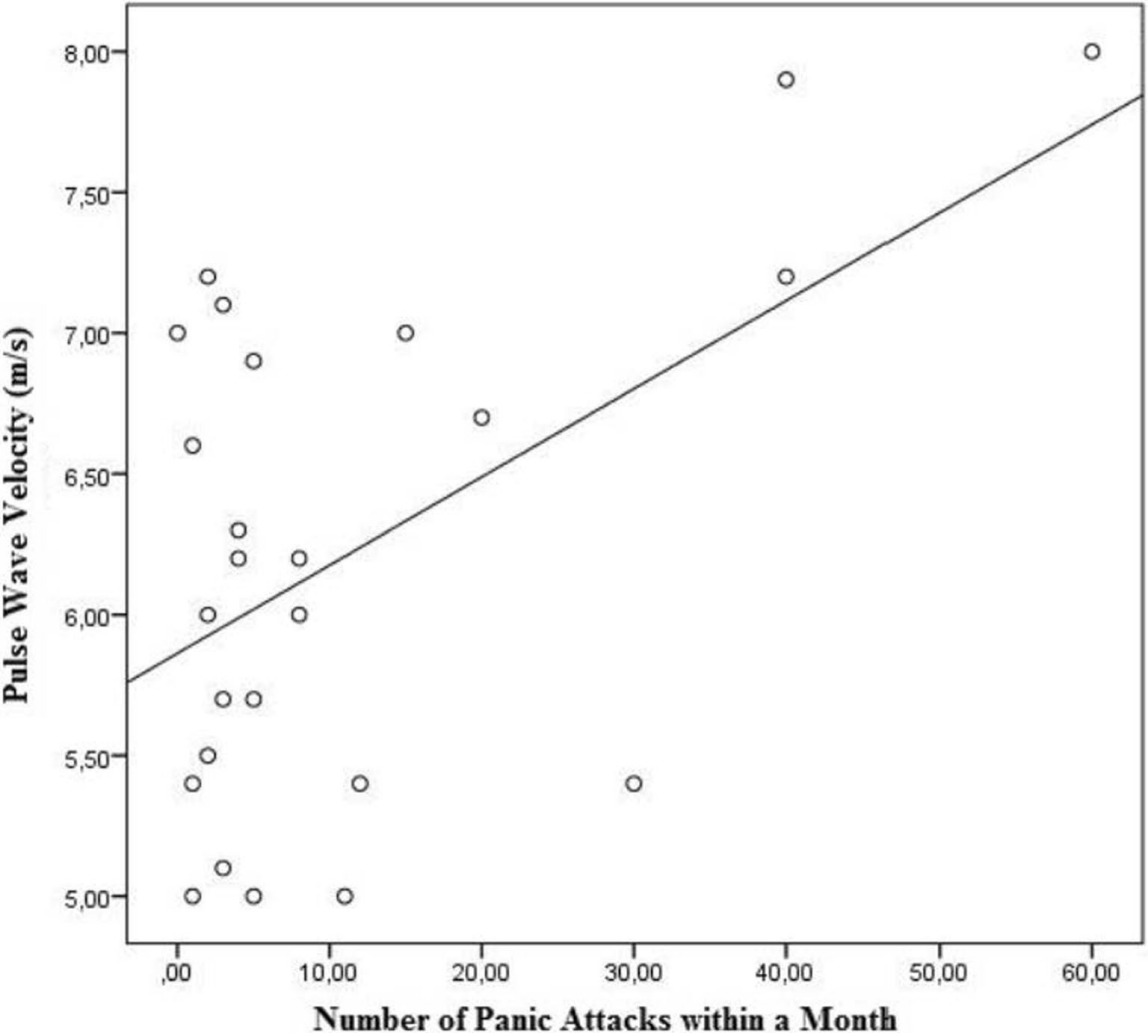
Correlation analysis of pulse wave velocity with number of attacks within a month in patients with panic disorder

Cardiac hemodynamic parameters are listed in Table 2. Peripheral and central systolic blood pressure were significantly higher in patients with panic disorder (*p* = 0.001, *p* = 0.002, respectively). Heart rate was also significantly higher in patients with panic disorder (*p* = 0.008). Comparison of PWV and AIx values of the study population are shown in Fig. 1. Patients with panic disorder had significantly higher PWV and AIx values compared to controls (*p* = 0.001, 0.006, respectively). Correlation analysis of BDI and BAI scores with arterial stiffness and hemodynamic parameters are shown in Table 3. There was a moderate correlation between PWV and AIx with BAI scores (Fig. 2). AIx was also correlated with BDI scores (*r* = 0.415, *p* = 0.415). Peripheral and central systolic blood pressure were correlated with BAI score (*r* = 0.353, *p* = 0.012, *r* = 0.385, *p* = 0.006), respectively. Heart rate was correlated with both BAI and BDI scores. We also assessed the correlation between PWV and number of attacks within a month in patients with panic disorder, and found a moderate correlation was between PWV and number of attacks within a month in patients with panic disorder (*r* = 0.529, *p* = 0.007) (Fig. 3).

**Table 2:** Comparison of cardiac hemodynamic parameters of study population

	Patient with panic disorder (*n* = 25)	Control group (*n* = 25)	*p*
Peripheral systolic blood pressure^a^ (mmHg)	126.2 ± 12.8	115.1 ± 8.5	*0.001*
Peripheral diastolic blood pressure^a^ (mmHg)	80.4 ± 10.7	76.1 ± 9.6	0.139
Peripheral pulse pressure^b^ (mmHg)	45.5 ± 14.4	38.8 ± 10.5	0.067
Heart rate^a^ (beat/min)	85.2 ± 14.5	75.6 ± 10.0	*0.008*
Cardiac index^a^ (l/min^c^ 1/m^2^)	2.4 ± 0.5	2.3 ± 0.4	0.225
Central systolic blood pressure^a^ (mmHg)	115.9 ± 12.1	106.8 ± 6.6	*0.002*
Central diastolic blood pressure^a^ (mmHg)	82.1 ± 10.6	77.6 ± 9.7	0.122
Central pulse pressure^a^ (mmHg)	33.5 ± 10.3	29.2 ± 7.7	0.103
Reflecting magnitude^a^ (%)	60.6 ± 8.9	62.6 ± 10.4	0.460

Italic values indicate statistically significant (*p* < 0.05)

^a^Student *T* test was used

^b^Mann–Whitney *U* test was used

^c^Data are presented as mean ± standard deviation

**Table 3:** Correlation analysis of depression and anxiety scores with arterial stiffness and hemodynamic parameters

	Beck Depression Inventory		Beck Anxiety Inventory	
	*r*	*p*	*r*	*p*
Pulse wave velocity (m/s)	0.176	0.220	0.442	*0.001*
Augmentation Index (%)	0.415	*0.003*	0.441	*0.001*
Peripheral systolic blood pressure (mmHg)	0.191	0.183	0.353	*0.012*
Central systolic blood pressure (mmHg)	0.150	0.299	0.385	*0.006*
Heart rate (beat/min)	0.351	*0.013*	0.365	*0.009*

Italic values indicate statistically significant (*p* < 0.05)

Linear regression analysis was performed to determine the predictors of BAI and BDI scores in patients with panic disorder. Among age, PWV, AIx, peripheral systolic blood pressure, and heart rate, which were included in the linear regression model, PWV was shown to significantly associated with BAI score in patients with panic disorder (r^2^ of the model = 0.424; *p* < 0.001) (Table 4).

**Table 4:** Linear regression analysis to determine the independent predictors of beck depression and beck anxiety scores

	BAI scores			BDI scores		
	*β*	*t*	*p*	*β*	*t*	*p*
Age	−0.386	−2.855	*0.007*	−0.229	−1.481	0.146
Peripheral systolic blood pressure	0.018	0.133	0.895	−0.020	−0.125	0.901
Heart rate	0.260	1.977	0.054	0.248	1.651	0.106
Pulse wave velocity	0.501	3.166	*0.003*	0.131	0.723	0.473
Augmentation Index	0.159	1.148	0.257	0.284	1.793	0.080

Italic values indicate statistically significant (*p* < 0.05)

*BAI* Beck Anxiety Inventory, *BDI* Beck Depression Inventory

## Discussion

Our study has revealed that hemodynamic parameters are deteriorated in patients with panic disorder in accordance with previous study [22]. This result may be related to autonomic hyperactivity, which is an important symptom cluster of panic disorder, or unhealthy lifestyle habits, which may be observed in patients with mental disorders [22]. The relationship between autonomic hyperactivity and hypertension, tachycardia, atrial fibrillation has been demonstrated in the studies and so, some of the treatments such as ganglion plexus ablation, renal sympathetic denervation are performed for reducing these complications [23, 24].

We have found significantly increased PWV and AIx scores in patients with panic disorder compared to control groups despite being in the maintenance period. This is in accordance with previous studies [8, 11] despite having relatively small sample and confounding factor such as being under antidepressant treatment. There are controversial results related to use of antidepressant agents and stiffness parameters. In a study use of duloxetine was related to increased PWV, however, this relation was not determined in escitalopram in older participants [25]. Therefore, the association between increased stiffness parameters and panic disorder might not be attributed exclusively to the panic symptoms, while antidepressant use may themselves influence these associations. Thus, comparing the data of antidepressant subgroups would be more explanatory. Nevertheless, the small sample size of this study did not allow such comparison. After all, arterial stiffness is related to stroke and cardiovascular mortality and patient with panic disorder might be under the risk of cardiovascular complications.

In a previous study Oulis et al. [13] have revealed that 6 week antidepressant treatment has significantly reduced the stiffness parameters in patients with depression compared to the control group. In this study researchers have demonstrated that full responders to antidepressant treatment have significantly greater vascular improvement than partial responders [13]. Although our clinical sample differs from Oulis et al. [13], we consider that only antidepressant treatment could not improve arterial stiffness due to having high scores of both BDI and BAI than control groups. Our results have demonstrated a positive correlation between BAI and two stiffness parameters (PWV, AIx), respectively, despite being under treatment. Seldenrijk et al. [8] has shown that increased arterial stiffness parameters in patients with depression and anxiety disorder result from symptom severity rather than using antidepressant medication. Our results are in accordance with Seldenrijk et al. [8]. Interestingly, we have found that BAI was correlated AIx and PWV (Fig. 2), however, BDI was correlated only AIx. These results may be related to the clinical features of panic disorder, which is a subgroup of anxiety disorders, or relatively small number of our clinical sample.

We have assessed the frequency of panic attacks by asking participants in the last month to demonstrate the attack and stiffness relationship, currently.

In literature, the knowledge is lacking about the relationship between frequency of panic attack and PWV. Here, we detected the mean number of panic attacks in a month by self-reports of the patients although this approach may be a little bit doubtful because of bias of recall. There was a positive correlation between PWV and the frequency of panic attacks. In the literature we could not detect any data about this relationship but Cicek et al. [11] has emphasized the lack of information regarding this relationship as a limitation to in their study. Given that the characteristic features of panic attacks are tachycardia, sweltering and other autonomic hyperactivity symptoms [26] then the frequency of panic attacks might be important for vascular health.

Although patients with panic disorder have significantly higher systolic blood pressure and heart rate than controls, their values of blood pressure and heart rate remain in normal range. There are several explanations for this reason. First, small sample size may lead to normal results in these cohorts. However, large scale studies demonstrated that patients with anxiety disorders have higher blood pressure values [27, 28]. Second, we only measured the blood pressure values of study population during assessment of arterial stiffness parameters. However, previous studies have demonstrated that, nocturnal and early morning hypertension are also associated with anxiety disorder [28, 29].

Our study has some limitations, which can be listed as; being a small sample, cross sectional design, unable to control confounding factors including life style, family history, SSRI use, variability of SSRI medications (i.e., fluoxetine and sertraline), variations in the duration of antidepressant usage and self report nature of panic attack assessment which is subject to recall bias. In addition, we only measured blood pressure value during assessment of arterial stiffness parameters. If we had 24 h blood pressure monitoring of study population, possibly we may reach to clinically significant results in these cohorts.

## Conclusion

Due to the cross sectional design, we were unable to demonstrate precise mechanism of increased arterial stiffness in panic disorder patients and we have shown significantly increased arterial stiffness parameters in PD. We revealed positive correlation between anxiety and depression scores and arterial stiffness parameters in patients with PD despite relatively small size of our study. The frequency of panic attacks may be important for arterial stiffness. The relationship between antidepressant treatment and panic attack, symptom severity is not elucidated. Thus, further longitudinal studies are needed, related to stiffness, panic disorder, symptom severity, for the clarification of this association in drug naive patients.

## Key points

- Determining the relationship between panic disorder and cardiovascular disease may be an important issue.
- We have shown an association between arterial stiffness parameters and depression/anxiety scores in patients with panic disorder under treatment period.
- Patients with panic disorder, despite being under-treatment, appear to have worse stiffness parameters than the healthy control group.
- Increasing levels of anxiety and depression may be related to worsening stiffness parameters despite confounding factors of our study.
